# Blood DNA methylation of *EIF5A* and *TGIF1* is associated with adipose tissue health and metabolic outcomes in obesity: a multi-cohort study

**DOI:** 10.1186/s13148-026-02177-y

**Published:** 2026-06-16

**Authors:** Sophie Ruf, Anne Hoffmann, Luise Müller, Kathrin Landgraf, Mandy Vogel, Emil Jørsboe, Phil Kubitz, Arne Dietrich, Adhideb Ghosh, Falko Noé, Christian Wolfrum, Melina Claussnitzer, Hans Hauner, Michael Stumvoll, Ronny Baber, Matthias Blüher, Antje Körner, Peter Kovacs, Maria Keller

**Affiliations:** 1 Medical Department III - Endocrinology, Nephrology and Rheumatology, Leipzig University, Liebigstraße 19, 04103 Leipzig, Germany; 2 Helmholtz Institute for Metabolic, Obesity and Vascular Research (HI-MAG), Helmholtz Munich at Leipzig University and University of Leipzig Medical Center, Philipp-Rosenthal Str. 27, 04103 Leipzig, Germany; 3 Center for Pediatric Research, Leipzig University, Medical Faculty, University Hospital for Children and Adolescents, Leipzig, Germany; 4 German Center for Child and Adolescent Health (DZKJ), Partner Site Leipzig/Dresden, Leipzig, Germany; 5 LIFE - Leipzig Research Centre for Civilization Diseases, Leipzig University, Leipzig, Germany; 6 Nuffield Department of Population Health, University of Oxford, Oxford, UK; 7 Big Data Institute, Li Ka Shing Centre for Health Information and Discovery, University of Oxford, Oxford, UK; 8 Faculty of Health and Medical Sciences, Novo Nordisk Foundation Center for Basic Metabolic Research, University of Copenhagen, Copenhagen, Denmark; 9 Center for Liver Research, Department of Gastroenterology and Hepatology, Odense University Hospital, Odense, Denmark; 10 Else Kröner-Fresenius-Center for Nutritional Medicine, Chair of Nutritional Medicine, School of Life Sciences, Technical University of Munich, Freising-Weihenstephan, Germany; 11 Institute for Nutritional Medicine, School of Medicine and Health, Technical University of Munich, Munich, Germany; 12 Medical Department of Visceral, Transplantation, Thoracic and Vascular Surgery, University of Leipzig, Leipzig, Germany; 13 Institute of Food, Nutrition and Health, ETH Zürich, Schwerzenbach, Switzerland; 14 Medical and Population Genetics Program & Broad Diabetes Initiative, Broad Institute of MIT and Harvard, Cambridge, MA USA; 15 Novo Nordisk Foundation Center for Genomic Mechanisms of Disease, Broad Institute of MIT and Harvard, Cambridge, MA USA; 16 Diabetes Unit and Center for Genomic Medicine, Massachusetts General Hospital, Boston, MA USA; 17 LeiCeM - Leipzig Center of Metabolism, Leipzig University, Leipzig, Germany; 18 Institute of Laboratory Medicine, Clinical Chemistry, and Molecular Diagnostics, Leipzig University, Leipzig, Germany; 19 German Center for Diabetes Research (DZD), Neuherberg, Germany

**Keywords:** DNA methylation, mRNA expression, Epigenetics, Childhood, Obesity, Adipose tissue, Blood, Metabolism

## Abstract

**Background:**

Blood-based DNA methylation has been linked to obesity and metabolic health, yet its relationship to adipose tissue function remains incompletely understood. This study aimed to investigate the epigenetic regulation of *EIF5A (Eukaryotic translation initiation factor 5A-1*) and *TGIF1 (TGFB Induced Factor Homeobox 1)* across blood and adipose tissue in obesity.

**Methods:**

Candidate genes were identified using a multi-step gene selection approach integrating transcriptomic and epigenomic data from blood and adipose tissue samples obtained from children and adults across four diverse population- and disease-focused cohorts. Genes were prioritized based on differential DNA methylation and gene expression in obesity. Targeted bisulfite sequencing of *EIF5A* and *TGIF1* was conducted in blood samples from adults across BMI-defined groups and from children prior to the development of obesity, to validate candidate loci and to examine associations with metabolic and adipocyte-related phenotypes.

**Results:**

In adults from the Leipzig Obesity BioBank, blood DNA methylation (*N* = 150) of both *EIF5A* and *TGIF1* was significantly increased in individuals with obesity. *EIF5A* mRNA expression (*N* = 1554) was significantly higher in omental-visceral adipose tissue compared with subcutaneous adipose tissue. Blood DNA methylation of *EIF5A* was associated with body mass index (BMI), glycated hemoglobin (HbA1c), and leukocyte counts, particularly among individuals with type 2 diabetes mellitus. In children, blood DNA methylation (*N* = 75) of *EIF5A* was associated with longitudinal HbA1c trajectories. For *TGIF1*, DNA methylation levels were increased in subcutaneous adipose tissue (*N* = 219) of children with obesity and correlated with fasting serum insulin concentrations. Across cohorts, *TGIF1* regulation in both blood and adipose tissue showed consistent associations with adipocyte size. Notably, blood DNA methylation of *TGIF1* in childhood was associated with body fat mass and HbA1c at later follow-up despite normal weight at baseline.

**Conclusion:**

*EIF5A* and *TGIF1* DNA methylation represent cross-tissue epigenetic signatures linking blood-based DNA methylation to adipose tissue dysfunction, adipocyte hypertrophy, and early metabolic risk. These findings support the potential of blood DNA methylation markers to reflect adipose tissue health and metabolic outcomes across the lifespan.

**Supplementary Information:**

The online version contains supplementary material available at 10.1186/s13148-026-02177-y.

## Background

Obesity is a worldwide epidemic challenging the health care systems by increasing the risk of various diseases such as cardiovascular diseases (coronary heart disease, hypertension, stroke), certain types of cancer, Type 2 Diabetes mellitus (T2D), gout or obstructive sleep apnea [1, 2]. Consequently, obesity goes along with reduced life expectancy and higher hospitalization costs [3]. Thus understanding its genetic and environmental determinants is crucial for effective prevention and treatment of related conditions [4]. Various studies have demonstrated the role of genetics in obesity [5–8], with twin studies suggesting that up to 70% of BMI variance can be attributed to genetic factors [9]. However, it is not only gene mutations or single nucleotide polymorphisms (SNPs) that may increase susceptibility to develop obesity (the combined effect of all SNPs only explains about 6% of BMI variance [8, 10]), but also by more subtle epigenetic mechanisms such as DNA methylation (DNAm), which can modulate gene expression [11]. Indeed, adipose tissue (AT) may retain an epigenetic memory that persists even after weight loss [12].

DNAm patterns are tissue specific [13] and can be influenced by lifestyle factors such as exercise, smoking, and alcohol consumption [14]. Studies investigating lifestyle-associated interventions such as exercise, diet and bariatric surgery for weight loss detected altered methylation patterns in various tissues such as AT, muscle, and blood [15–20]. This is particularly relevant, as such epigenetic changes may not only reflect short-term metabolic adaptations but could also contribute to a lifelong predisposition to obesity and might even influence the health of subsequent generations through transgenerational epigenetic inheritance [12, 21–25]. Methylation patterns may thus represent the consequence of an interaction between genetic predisposition and environmental effects [26–28]. Although, some epigenetic biomarkers are already identified and used in oncology or neurology [29–33], they are not yet used as stratification markers for metabolic diseases [34]. However, various methylation-based risk scores with potentially prognostic insights were recently developed. One was able to outperform conventional risk scores in predicting diabetes onset [11], one risk score for T2D showed to be associated with gestational diabetes [35], while another study with almost 10,000 participants showed that the individual habitual diet is associated with the methylation of 30 specific CpG-sites, cardiovascular risk, and increased all-cause mortality [36]. These examples provide evidence for the need of stable, easy to access epigenetic biomarkers to stratify metabolic states in obesity. In line, it was recently successfully demonstrated that although DNAm is to a large extent tissue specific, blood can be used to reflect epigenetic remodeling of inflammatory pathways in AT after bariatric surgery induced weight-loss [37]. Epigenome-wide association studies (EWAS) investigating obesity and related phenotypes often identify a large number of candidate genes [38, 39], making it challenging to pinpoint those most relevant for further clinical validation. Moreover, such approaches typically do not integrate gene expression data and therefore provide limited insight into whether methylation changes relate to transcriptional regulation. To overcome this, in the present study, we analyzed four different cohorts using different omic (DNAm/mRNA expression) as well as cross-tissue datasets (blood/ AT) to identify candidate genes consistently altered between individuals with and without obesity with the overarching aim to identify potential, clinically accessible blood derived biomarkers. The two identified genes, *EIF5A (Eukaryotic translation initiation factor 5A-1*) and *TGIF1 (TGFB Induced Factor Homeobox 1)* were followed up to investigate their epigenetic role in obesity and their diagnostic potential as early biomarkers of metabolic dysfunction. Here, we move beyond conventional single-tissue EWAS by applying a cross-tissue, cross–life-stage integrative framework that systematically links blood DNAm to AT transcriptional and epigenetic alterations. This approach enables the prioritisation of blood-based DNAm loci that reflect AT health and metabolic dysfunction, thereby enhancing biological interpretability and translational relevance.

## Methods

### Study design and characteristics

**Fig. 1:**
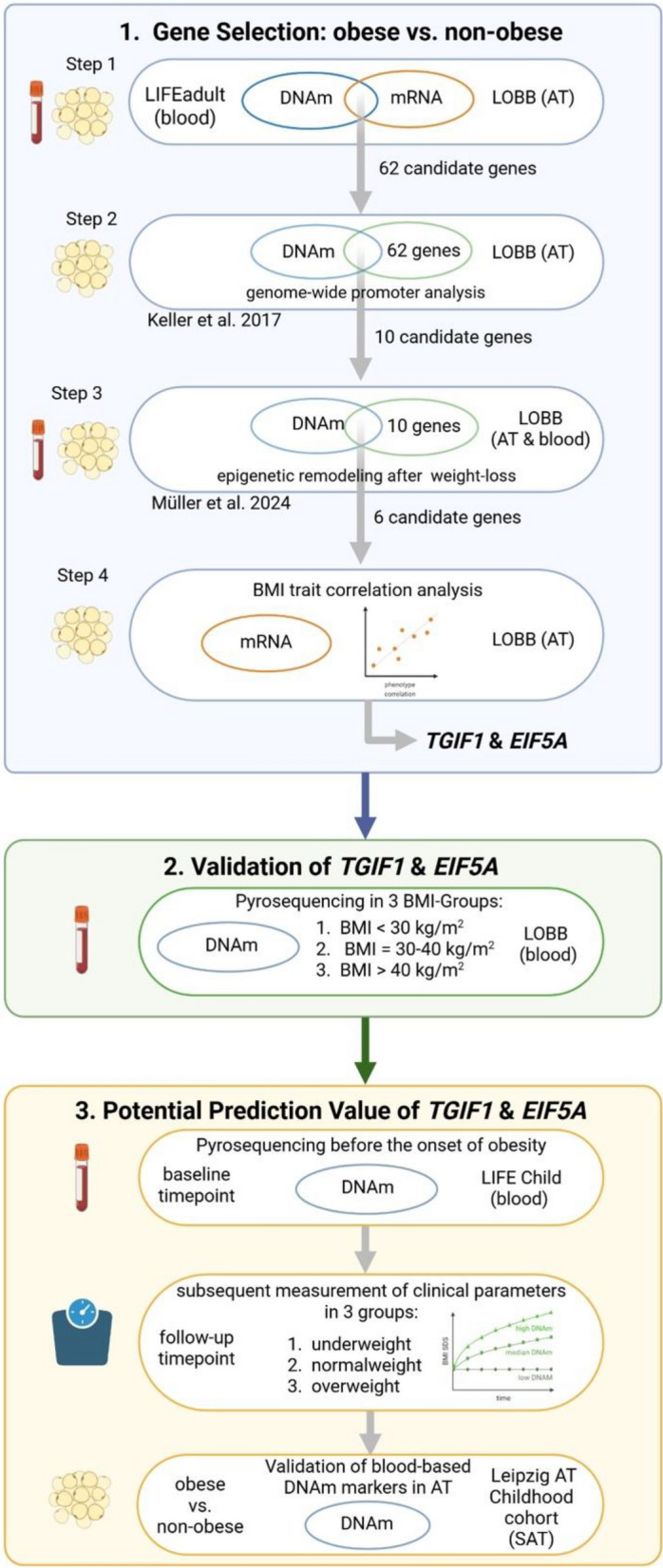
Workflow scheme for the identification (blue box), validation (green box), and predictive assessment (yellow box) of candidate genes associated with obesity. *Created with BioRender.com.* Gene Selection process (blue box): *Step 1:* DNAm (DNA methylation indicated by a blue circle) from the LIFE-Adult cohort (in blood – the used tissues are shown on the left [14]) and mRNA expression data (orange circle) from the LOBB (LeipzigObesityBioBank) cohort (in AT—adipose tissue) were integrated to identify genes which are differentially regulated between obese and non-obese individuals. 62 candidate genes were identified having both: differential DNAm and mRNA expression pattern, leading to *Step 2* (arrow): These selected 62 genes (green circle) were cross-referenced with DNAm data in AT (indicated by schematic picture on the left) of promoter regions from Keller et al. [41], narrowing the list to 10 candidates (arrow). *Step 3:* Further filtering of the candidate genes based on DNAm remodeling in blood and AT (shown on the left), associated with weight loss after bariatric intervention in Müller et al.[37], resulted in 6 genes (arrow). *Step 4:* Correlation analysis between mRNA expression in AT (shown on the left) of the LOBB cohort and clinical characteristics identified *TGIF1* and *EIF5A* as the final candidate genes (in bold). Validation process (depicted below in a green box): DNAm (blue circle) levels of *EIF5A* and *TGIF1* were validated in blood samples (shown on the left) of the LOBB cohort using targeted pyrosequencing, showing significant differences in DNAm across different BMI (body mass index) groups (< 30/ 30–40/ > 40 kg/m^2^). Process of Predictive Value assessment of both genes (yellow box): Step 1: DNAm (blue circle) of *EIF5A* and *TGIF1* was measured in blood (shown on the left) in children of the LIFE Child cohort at a timepoint with all children having normal weight (T0) according to BMI SDS. Step 2: Children were phenotyped and stratified into groups with underweight, normal weight or overweight (based on BMI SDS) at a later timepoint (T1). DNAm at T0 was correlated with clinical characteristics at T1. Step 3 (not depicted): Additionally, DNAm was validated in SAT (subcutaneous adipose tissue) in children with and without obesity of the Adipose Tissue Childhood cohort and correlated with several phenotypes at the same timepoint

The study design, illustrated in Fig. 1, follows a multi-step selection process aimed at identifying the most promising blood DNAm markers that reflect or potentially predict AT health and metabolic function, integrating data from four cohorts: the LIFE-Adult study [40], the Leipzig Obesity BioBank (LOBB) [37, 41, 42], the Leipzig Adipose Tissue Childhood Cohort [43] and the LIFE-Child study [44].

The LIFE-Adult Study is a population-based cohort with more than 10,000 participants from Leipzig (Germany), which is intensively phenotyped [40, 45]. Among them we included DNAm (Illumina EPIC arrays) data of blood from *N* = 100 individuals in our multi-step selection analysis (Fig. 1 – step 1). Those individuals consist of 56 females and 44 males with a mean age of 57.8 ± 6.2 years and an average BMI of 28.6 ± 6.5 kg/m^2^. 50 individuals were either without or with obesity [14] and all study characteristics are described in Supplemental Table 1 (DNAm discovery cohort).

The LOBB includes over 8,500 participants, comprising individuals with obesity as well as healthy volunteers who donated biomaterials such as blood as well as paired subcutaneous adipose tissue (SAT) and omental-visceral adipose tissue (OVAT) samples. LOBB measures a comprehensive range of anthropometric traits and metabolic variables, collected as described previously [42, 46], while AT samples were collected during elective laparoscopic abdominal surgeries as shown in earlier studies [47]. Bulk RNA-seq transcriptome data from SAT and OVAT were available for 1,554 individuals (70% females; mean ± SD: age 48.1 ± 12.6 years; BMI 47.7 ± 9.8 kg/m^2^, comprising 83 individuals classified as non-obese and 1,471 as obese) and were incorporated into our multi-step candidate gene selection process (Fig. 1 – step 1 and 4). Details are described in Supplemental Table 2 (mRNA discovery cohort).

From the LOBB, a subset of 77 individuals (44 females, 33 males; 36 with and 41 without obesity) with genome-wide DNA promoter methylation data from SAT and OVAT obtained during abdominal surgery [41] was included in the selection process (Fig. 1 – step 2). These participants had a mean age of 59.8 ± 10.3 years and a mean BMI of 31.1 ± 11.5 kg/m^2^ (details shown in Supplemental Table 3).

Another subset of nine individuals [37] of LOBB with severe obesity who underwent two-step bariatric surgery were also included (Fig. 1 – step 3). Selection criteria required availability of blood, SAT, and OVAT samples before and after bariatric surgery and a minimum of 30% excess BMI reduction by the second surgery. This group included six females and three males with a mean age at the first surgery of 52.2 ± 13.3 years and an average interval of 3.0 ± 2.3 years between the surgeries. The mean BMI was 54 ± 9.3 kg/m^2^ before the first surgery and 40.9 ± 4.5 kg/m^2^ before the second surgery (details described in Supplemental Table 4).

A final subset of 150 LOBB participants was newly selected for the validation of the identified candidate loci using DNA from EDTA blood samples (Fig. 1 – part 2: Validation), of which 118 individuals also had matching RNA-seq data. The validation cohort included 87 women and 63 men, with a mean age of 49.6 ± 11.9 years and an average BMI of 37.5 ± 9.9 kg/m^2^. Subjects were matched for age and sex as closely as possible. Among them, 63 were diagnosed with T2D according to the guidelines of the American Diabetes Association. The 150 participants were stratified into three BMI groups: normal/overweight (BMI < 30, N = 50), obese (BMI 30–40, N = 50), and severely obese (BMI > 40, N = 50). Phenotypic characteristics are summarized in Supplemental Table 5.

The LIFE-Child study is a population-based longitudinal study in Leipzig examining the period from pregnancy to adulthood to provide insight into the development of children [44]. More than 3,000 children took part by the end of 2015, and two thirds of them continuously. The study consists of three linked cohorts: the birth cohort, the health cohort and the obesity cohort [44, 48–50]. We employed a longitudinal study design using two timepoints to analyse predictive values. At the first timepoint, blood samples were collected from a group of *N* = 75 children (39 males and 36 females), who were all classified as normal weight at that time. At the time of blood sampling, the children were 8.6 ± 2.7 years old and had a BMI SDS (Standard Deviation Score [43, 48, 49]) of 0.07 ± 0.74. At a subsequent timepoint, a BMI assessment was conducted, and children were selected based on their BMI classification at that time. Specifically, 39 children were classified as normal weight, while 19 children were categorized as underweight and 17 children as overweight. The average age at this second timepoint was 16.4 ± 1.8 years with an average time between blood sampling and the follow-up BMI state assessments of 7.8 ± 3.2 years (phenotypical characteristics shown in Supplemental Table 6). This cohort was used to evaluate the selected genes as predictive marker in children (Fig. 1 – part 3: Predictive Value).

Leipzig Adipose Tissue Childhood Cohort comprises AT samples from 219 children with (N = 77) and without (N = 142) obesity, defined by the BMI SDS according to the German Working Group for Paediatric Consensus Guidelines [51, 52]. The mean BMI SDS was -0.07 ± 0.95 for the group without and 2.49 ± 0.53 for the group with obesity, with an average age of 10 ± 5.3 years across all participants. Of the 219 children 88 were females and 131 were males. Only SAT samples, obtained during elective surgeries were used [43]. In this cohort, an epigenome-wide methylation analysis was performed (Illumina HumanHT-12 v4 BeadChip arrays), enabling the assessment of the entire gene region rather than a limited number of CpG sites, in contrast to the targeted bisulfite sequencing applied to the blood samples of the LOBB and LIFE Child cohort. This cohort was used to validate blood-based DNAm markers by comparing them with AT patterns in children (details described in Supplemental Table 7).

### Bulk RNA sequencing and analysis

Library preparation and RNA-seq data processing of the LOBB samples of SAT and OVAT were performed as previously described [53]. In brief, RNA was extracted from AT samples using the SMARTseq protocol. Libraries were prepared and sequenced (single-end mode) on a NovaSeq 6000 platform at the Functional Genomics Centre Zurich. After adaptor and quality trimming, reads were aligned to the human reference genome (GRCh38.p13; GENCODE v32), and gene-level abundances were quantified using Kallisto (v0.48) [54]. Samples exceeding 20 million reads were down sampled to this depth with the R package ezRun (v3.14.1) [55]. Expression values were normalized using the variance stabilizing transformation (VST) method and adjusted for age, transcript integrity number, and sex. Differential Gene Expression (DGE) analysis between individuals without (BMI < 30 kg/m^2^) and with (BMI ≥ 30 kg/m^2^) obesity were conducted using the R package DESeq2 (v1.32.0) [56]. Differentially expressed genes (DEGs) with an adjusted P < 0.01 were considered statistically significant.

### Genome-wide DNA methylation and analysis

Blood samples were collected, processed, and bisulfide converted following established protocols described previously [14]. After quality assessment, DNA methylation was measured across the genome using Illumina HumanMethylation850 BeadChips (Illumina, San Diego, USA). The iScan platform was used for array imaging and quantification in collaboration with the Core Unit DNA technologies at the University of Leipzig and GenomeScan in Leiden, Netherlands, yielding DNAm levels for ~ 850,000 CpG loci per sample at single-nucleotide resolution. Raw methylation data were imported, pre-processed, and normalized using the ChAMP R package (v2.26.0) [57], applying beta-mixture quantile normalization (BMIQ). Probes failing detection (P > 0.01) in > 1% of samples, those with fewer than three beads in ≥ 5% of samples, non-CpG probes, cross-reactive probes, and probes containing known SNPs at the CpG-sites were excluded based on established annotations [58]. Probes on sex chromosomes (X, Y) were also removed to minimize sex-related variation. After filtering, 709,855 probes across 98 samples remained for the LIFE-Adult cohort and 738,022 probes across 219 samples for the Leipzig Adipose Tissue Childhood Cohort. Cell-type heterogeneity was corrected using the reference-based method implemented in ChAMP. Data were adjusted for age, sex, array, and slide (LIFE-Adult) or for age, sex, array, and run (Leipzig Adipose Tissue Childhood Cohort). Using the mCSEA [59], differentially methylated regions (DMRs) between individuals without (BMI < 30 kg/m^2^) and with (BMI ≥ 30 kg/m^2^) obesity in the LIFE-Adult cohort (Fig. 1, step 1) were identified, with a focus on gene body and promoter regions comprising at least three CpG sites. DMRs with an adjusted P < 0.01 were considered statistically significant.

### Candidate gene selection

As shown in Fig. 1 we performed a multi-step selection process to identify the most promising blood DNAm markers, discriminating patients with (BMI > 30 kg/m^2^) from patients without (BMI < 30 kg/m^2^) obesity, which most likely also mirror target tissue specific function.

To do so we initially (**step 1**) conducted DEGs from the genome-wide transcriptome data of AT from LOBB as well as the DMRs from genome-wide methylome data of blood of the LIFE-Adult cohort [14]. This analysis was performed separately in the promoter region and in the gene body region for the DNAm as well as it was divided between the different fat types (SAT and OVAT) for the transcriptome data. Results from the four analyses with an adj. P below 0.01 were overlayed in terms of gene IDs which fulfil both: differential methylation as well as differential expression. These overlaying genes were taken forward to the next step. For each of the four analyses (gene body or promoter combined with each SAT or OVAT) the differential expression/methylation can be upregulated or downregulated, leaving 4 additional combinations. Subsequently, all potential combinations were grouped, resulting in 16 groups. From each of the 16 groups, the top 5 candidate genes (where available) were chosen based on the greatest disparity (between individuals with and without obesity) in DNAm, followed by expression, leading to 62 candidate genes (Supplemental Table 8) for further investigation. Within **step 2** (Fig. 1), we replicated these candidate genes using previously published data of AT DNAm in patients with and without obesity[41] in a subset of the LOBB cohort, expanding beyond our initial blood-based analyses. Genes which were also epigenetically regulated in AT were taken forwards to the next step, leading to 10 candidate genes. In **step 3**, we further validated these 10 genes in a recently published subset cohort from the LOBB, which included matched blood, SAT, and OVAT samples from the same patients before and after bariatric surgery-induced weight loss[37]. This step uniquely allowed us to assess dynamic changes in DNAm in response to weight loss, ultimately identifying six candidate genes that showed differential DNAm following the intervention, namely: *TGIF1* (TGFB induced factor homeobox 1)*, EIF5A* (Eukaryotic translation initiation factor 5A), *FAF1* (Fas associated factor 1), *ZBTB20* (Zinc finger and BTB domain containing 20), *DST* (Dystonin) *and HDAC4* (Histone deacetylase 4). To further investigate the molecular role of our final list of six genes*,* we performed Pearson correlation analyses between gene specific mRNA levels in the initial AT and the relevant metabolic traits in LOBB. Results were corrected for multiple testing via Benjamini–Hochberg correction [60] and utilized for the selection process (Supplemental Table 9). Finally, we reviewed the existing literature on the candidate genes to further substantiate the selection of *EIF5A* and *TGIF1* for in vivo validation using targeted bisulfite sequencing (selection process descripted more detailed in Supplemental Appendix). As both genes were identified in the primary analysis as hypermethylated and downregulated in individuals with obesity, regions within their promoters were chosen for targeted bisulfite sequencing.

### Targeted DNA methylation analysis

Blood samples were collected following an overnight fast. DNA was extracted using the DNA Extractor® SP Kit Maxi (WAKO Chemicals, Richmond, VA, USA) for LOBB samples and the Autopure LS platform (Qiagen, Hilden, Germany) for LIFE cohort samples, according to the manufacturers’ protocols. DNA concentration was measured using a NanoDrop 2000 spectrophotometer (Thermo Scientific, Waltham, MA, USA) and the Quantus fluorometer (Promega, Madison, WI, USA), and DNA was stored at − 80 °C until further use.

For bisulfite conversion, 500 ng of DNA per sample were treated using the EpiTect Fast DNA Bisulfite Kit (Qiagen, Hilden, Germany). Target regions were amplified with PyroMark PCR Master Mix (Qiagen, Hilden, Germany) using self-designed primers flanking the regions of interest (Supplemental Table 10). PCR products were quality-checked via gel electrophoresis and subsequently pyrosequenced on a PyroMark Q24 system with Gold Q24 reagents (Qiagen, Hilden, Germany). All measurements were performed in duplicate, with two non-template controls included in each run. Results were analyzed using PyroMark Q24 software, and low-quality samples were excluded. Samples showing > 10% deviation between duplicates were repeated once.

### Statistical analyses

Statistical analyses were conducted using the SPSS software (IBM Corp. Released 2021. IBM SPSS Statistics for Windows, Version 28.0. Armonk, New York, United States) or the statistical software R (R Core Team 2022, R: A language and environment for statistical computing, R Foundation for Statistical Computing, Vienna, Austria). Graphical figures were generated using GraphPad Prism (GraphPad Software version 10.0.0, Boston, Massachusetts, United States), the statistical software R and BioRender.com. Normality of the data distribution was assessed by Kolmogorov-Smirnoff test and not normally distributed data was logarithmic or exponentially transformed. The DNAm levels were analysed in relation to phenotypic variables and across different BMI groups, as well as in connection with the RNAseq data (LOBB) from AT for the targeted DNAm analysis in adults, if available. Non-parametric Kruskal–Wallis tests were employed to compare methylation across BMI groups, with Bonferroni correction applied for multiple group comparisons. If data exhibited normal distribution, partial Pearson correlation analyses were performed for the results of the targeted DNAm analysis between the methylation and phenotypes, with age, sex and BMI included as covariates where applicable. Non-partial Pearson correlation analyses were conducted to explore the relationship between transcription or methylation data and phenotypic variables, as the transcription data was adjusted for age, transcript integrity number (TIN) and sex. Additionally, linear regression models were employed between the DNAm of *EIF5A* and HbA1c of individuals either with normal glucose tolerance or with impaired glucose tolerance as well as T2D. Spearman correlation analyses, without adjustment for age and sex, were utilized for non-parametric data. For all correlations we performed a Benjamini–Hochberg correction [60]. Paired t-tests were utilized to assess expression differences between SAT and OVAT. To compare the different BMI-Groups in the several cohorts an ANOVA or T-test for equality of means was used for either two or three groups respectively. For the comparison of the phenotypes before and after bariatric intervention a Wilcoxon signed ranked test was applied.

## Results

### Identification and prioritization of candidate genes linked to obesity and metabolic traits

As shown in Fig. 1 (and described in detail in the methods section), we used a multi-step approach to identify blood DNAm markers that discriminate individuals with (BMI > 30 kg/m^2^) from those without (BMI < 30 kg/m^2^) obesity and may reflect AT specific regulation. **Step 1** (Fig. 1) combined genome-wide transcriptome data from SAT and OVAT in LOBB with blood methylome data from the LIFE-Adult cohort to identify genes showing both differential expression and differential methylation, resulting in 62 candidate genes (Supplemental Table 8). In **Step 2** (Fig. 1), these candidates were filtered using published AT methylation data (LOBB subset [41]), yielding 10 genes also epigenetically regulated in AT. **Step 3** (Fig. 1) validated these genes in another subset of LOBB patients with matched blood, SAT, and OVAT samples before and after bariatric surgery [37], allowing assessment of methylation changes with weight loss and identifying six final candidates: *TGIF1, EIF5A, FAF1, ZBTB20*, *DST* and *HDAC4.* Finally, we investigated potential associations with metabolic traits in AT, by conducting correlation analyses using mRNA levels of these candidates (Step 4—Fig. 1, Supplemental Table 9): In SAT, both the negative correlations of *TGIF1* mRNA expression with body fat percentage (r_Pearson_ = -0.13, P < 10⁻^4^) and with serum cholesterol levels (r_Pearson_ = -0.11, P < 10⁻^4^) remained significant after correction for multiple testing. Additional associations were observed for *HDAC4*, *DST*, and *ZBTB20* (Supplemental Table 9). In OVAT, the inverse correlation between *TGIF1* and mean adipocyte diameter (r_Pearson_ = -0.20, P < 10⁻^4^), as well as the positive correlations of *EIF5A* with body fat percentage (r_Pearson_ = 0.13, P < 10⁻^4^) and hip circumference (r_Pearson_ = 0.12, P < 10⁻^4^), all remained significant following multiple-testing correction.

In OVAT, *EIF5A* and *TGIF1* were the only genes that displayed significant associations with phenotypes beyond BMI and body fat, which served as final selection criteria. Based on these findings, *EIF5A* and *TGIF1* emerged as the strongest candidates for further functional investigation and validation.

### Validation of the identified DNAm biomarker in blood

**Fig. 2:**
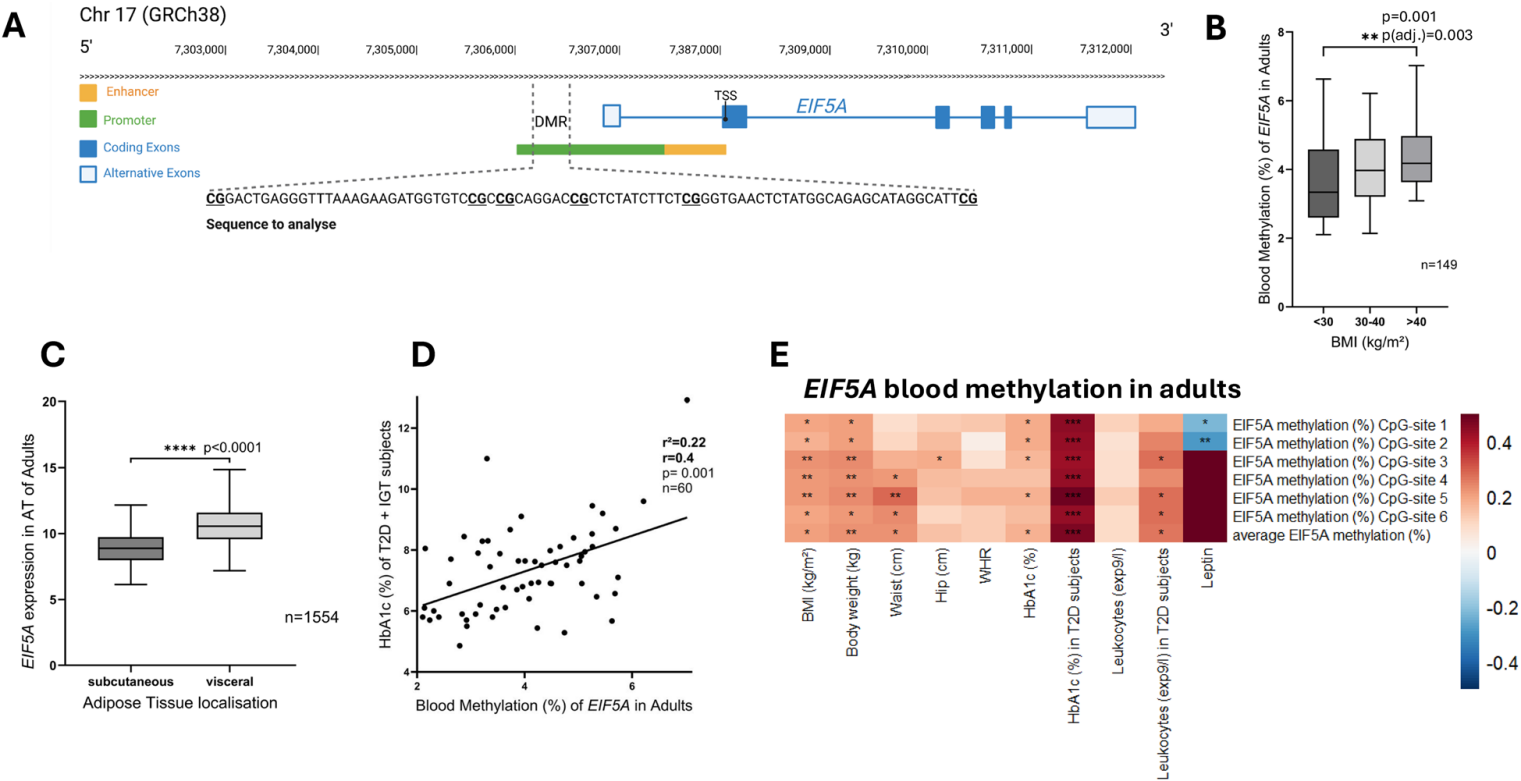
*EIF5A* regulation in adults (**A**) Genomic map of the *EIF5A* locus on chromosome 17p13.1 (GRCh38), indicating the region targeted by pyrosequencing (differentially methylated region, DMR). Methylation CpG-sites analysed in targeted pyrosequencing shown within the promoter-associated region and marked in bold and underlined. Exons are shown in blue, alternative exons in light blue. Regulatory elements include the promoter (green) and enhancer regions (yellow). TSS: Transcription Start Site. *Created with BioRender.com.* (**B**) Kruskal–Wallis test comparing the average percentage (DNAm levels represent the proportion of methylated cytosines at each CpG site in %) of *EIF5A* blood DNAm within the targeted region across BMI-defined groups (< 30 / 30–40 / > 40 kg/m^2^) in adults from the Leipzig Obesity BioBank (LOBB) validation cohort. P-values were adjusted for multiple testing using Bonferroni correction. N = 149. (**C**) Student’s t-test comparing *EIF5A* mRNA expression levels (log₂ VST) between subcutaneous (SAT) and visceral (OVAT) adipose tissue in adult LOBB participants. N = 1554. (**D**) Pearson correlation (r = 0.40) and linear regression between *EIF5A* blood DNAm (%) of targeted pyrosequencing and serum HbA1c (%) in adults with type 2 diabetes (T2D) or impaired glucose tolerance (IGT). N = 60. (**E**) Heatmap showing correlations between *EIF5A* blood DNAm at six CpG sites (%) analyzed by targeted pyrosequencing (and their mean DNAm) and various clinical characteristics in adults from the LOBB cohort. One asterisk (*) indicates p < 0.05, two asterisks (**) p < 0.01, and three (***) p < 0.001. None of the correlations remained significant after Benjamini–Hochberg correction for multiple testing. T2D: Type 2 Diabetes; WHR: Waist–Hip Ratio; BMI: Body Mass Index. Red indicates positive correlations and blue negative correlations, according to the legend

**Fig. 3:**
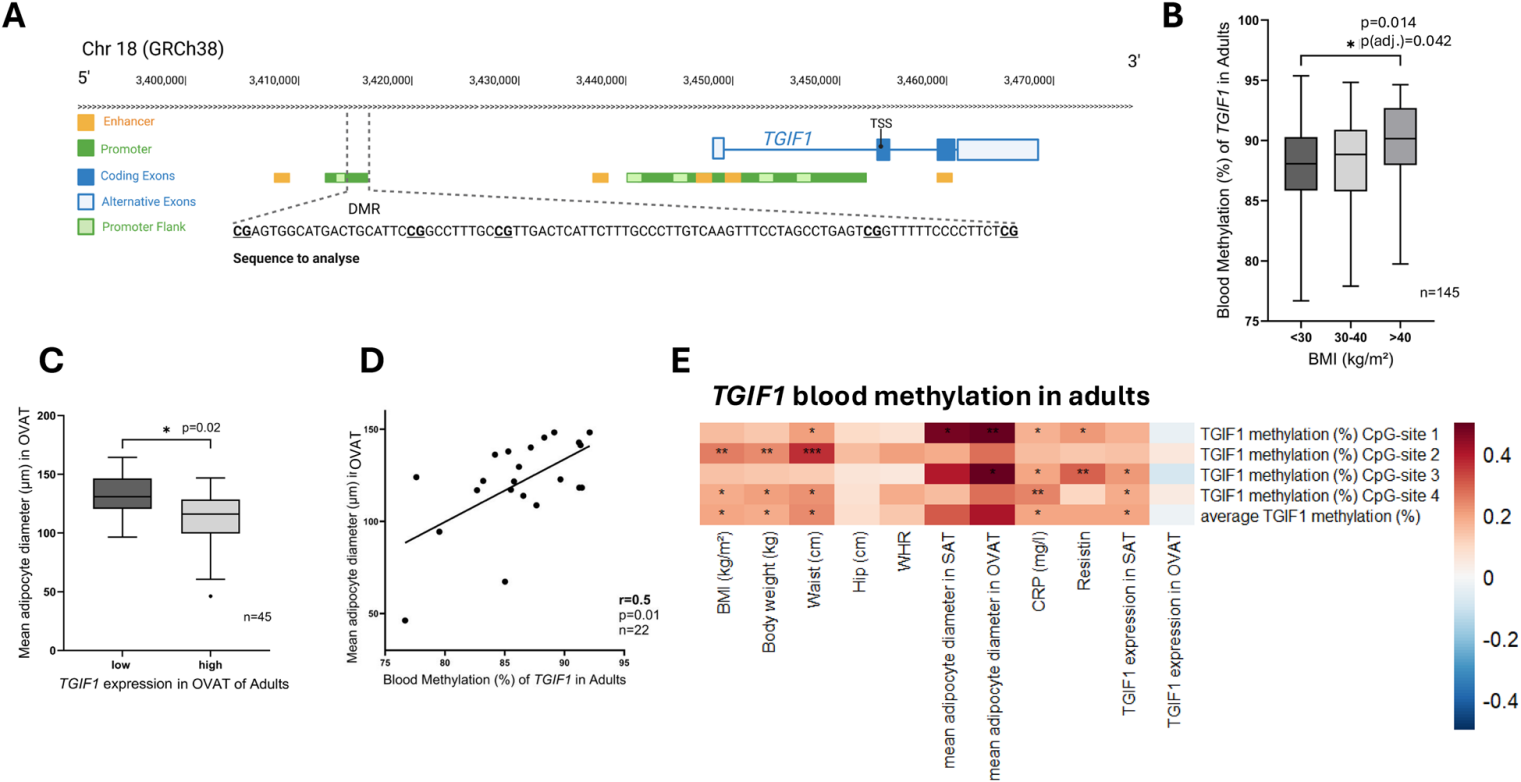
*TGIF1* regulation in adults (**A**) Genomic map of the *TGIF1* locus on chromosome 18p11.31 (GRCh38), indicating the region targeted by pyrosequencing (differentially methylated region, DMR). CpG sites analyzed in targeted pyrosequencing are shown within the promoter-associated region and marked in bold and underlined. Exons are depicted as blue boxes, alternative exons as light blue boxes. Regulatory elements relevant to transcriptional control are highlighted, including the promoter (green) and enhancer regions (yellow). TSS: Transcription Start Site. *Created with BioRender.com.* (**B**) Kruskal–Wallis test comparing the average percentage (%) of *TGIF1* blood DNAm within the targeted region of pyrosequencing across BMI-defined groups (< 30 / 30–40 / > 40 kg/m^2^) in adults of the Leipzig Obesity BioBank (LOBB) validation cohort. P-values were adjusted for multiple testing using Bonferroni correction. N = 145. (**C**) Student’s t-test comparing mean adipocyte diameter (µm) in visceral adipose tissue (OVAT) between participants in the lowest and highest decile of *TGIF1* mRNA expression (log₂ VST) in OVAT from adult LOBB participants. N = 45. (**D**) Spearman correlation (r = 0.50) with linear regression line between TGIF1 blood DNAm (%) analyzed in targeted pyrosequencing and mean adipocyte diameter (µm) in OVAT from adults in the LOBB study. N = 22. (**E**) Heatmap showing correlations between *TGIF1* blood DNAm at four CpG sites, and their average DNAm (%), analyzed with targeted pyrosequencing and various clinical characteristics in adults from the LOBB cohort. Clinical variables include body mass index (BMI), waist–hip ratio (WHR), C-reactive protein (CRP), waist circumference (Waist), hip circumference (Hip), and adipose-tissue measures from subcutaneous adipose tissue (SAT) and omental/visceral adipose tissue (OVAT). One asterisk (*) indicates p < 0.05, two asterisks (**) p < 0.01, and three (***) p < 0.001. Only the association between CpG-site 2 and waist circumference remained significant after Benjamini–Hochberg correction for multiple testing. Red indicates positive correlations and blue negative correlations, according to the legend

In Step 1 of our analysis, *EIF5A* and *TGIF1* were found to be hypermethylated and downregulated in individuals with obesity, prompting a targeted analysis of DNAm levels within their promoter regions. The genomic regions analyzed are located on chromosome 17 (GRCh38: 7,306,877–7,306,962) for *EIF5A* and chromosome 18 (GRCh38: 3,412,695–3,412,785) for *TGIF1*, as shown in Figs. 2A and 3A, respectively. We performed targeted bisulfide sequencing (pyrosequencing) for the *EIF5A* (chr17: 7,306,877 – 7,306,962) and *TGIF1* (chr18: 3,412,695 – 3,412,785) candidate regions (analogous to the regions identified by the EPIC data [37]) in a selected subset of the LOBB (BMI < 30 kg/m^2^/ BMI 30 – 40 kg/m^2^/ BMI > 40 kg/m^2^; *N*: 50/50/50). As expected, the groups showed significant differences in body weight (kg), bodyfat (%), waist und hip circumference (cm) as well as waist-to-hip-ratio (WHR) (all P < 0.05). Phenotypic characterisation as well as analysis of group differences are shown in Supplemental Table 5.

Validation of the DNAm profiles revealed a BMI group-specific increase for *EIF5A*. In detail, the average DNAm was 3.66% in individuals with BMI < 30 kg/m^2^, 4.00% in participants with BMI = 30-40 kg/m^2^ and 4.35% in those with BMI > 40 kg/m^2^, with an overall range from 2.1% to 7.0%. A Kruskal–Wallis test indicated statistically significant differences in DNAm between individuals of the non-obese and severely obese subgroup, which retained after Bonferroni correction (P-adj. = 0.003; Fig. 2B). No DNAm difference could be detected between both sexes nor between individuals with or without T2D. A correlation analysis between BMI and the mean DNAm across all *EIF5A* CpG-sites (Chr17: 7,306,877–7,306,962) confirmed a positive correlation (r_Spearman_ = 0.2, P = 0.01, Supplemental Table 11).

Similar to *EIF5A,* validation of *TGIF1* DNAm revealed a BMI-dependent increase in blood. We observed average DNAm levels at 87.5% in individuals with BMI < 30 kg/m^2^, 88.4% in those with BMI between 30 and 40 kg/m^2^, and 89.0% in individuals with BMI > 40 kg/m^2^ with an overall range of 70–95%. A Kruskal–Wallis test demonstrated statistically significant differences in mean DNAm levels between individuals without and with severe obesity, which remained significant after Bonferroni correction (P-adj. = 0.042; Fig. 3B). The average DNAm difference between these groups was 1.5% (SE = 0.9%). This trend of increased DNAm with higher BMI was consistent across both sexes. Notably, *TGIF1* DNAm levels did not differ significantly between individuals with or without T2D. A positive correlation between BMI and mean *TGIF1* DNAm across all CpG-sites (Chr18: 3,412,695–3,412,785) was observed (r_Spearman_ = 0.2, P = 0.02; Supplemental Table 11).

### *EIF5A* and *TGIF1* expression and methylation profiles are linked to metabolic traits

First, we detected significantly different expression levels for *EIF5A* between the OVAT and SAT (P < 1 × 10^–3^; Fig. 2C), with higher mRNA levels in OVAT. In line, we observed correlations between the mRNA levels in OVAT and body fat (in %, r_Pearson_ = 0.13, P < 0.001) and hip circumference (in cm, r_Pearson_ = 0.12, P = 0.002), which retained after correction for multiple testing (Supplemental Table 9). Furthermore, the HbA1c levels showed a significant correlation with the mean blood DNAm of *EIF5A* (r_Pearson_ = 0.17, P = 0.04). HbA1c and serum leucocyte count revealed a stronger correlation with the mean DNAm of all CpG-sites analyzed in *EIF5A* (Chr17: 7,306,877–7,306,962), when only testing the participants with impaired glucose tolerance and/or T2D (HbA1c: r_Pearson_ = 0.42, P = 0.001; Fig. 2D; Blood leucocyte count: r_Pearson_ = 0.3, P = 0.02), whereas no association could be observed in individuals with normal glucose tolerance. In this analysis, the results remained significant after excluding participants with impaired glucose tolerance. Additionally, the DNAm at CpG-site 3 of the designed assay (Chr17:7,306,909) correlated with hip circumference (r_Spearman_ = 0.2, P = 0.04). All correlations with DNAm are shown in Fig. 2E and Supplemental Table 11. 

Interestingly, *TGIF1* expression in SAT was positively correlated with DNAm at several CpG sites in blood, including a notable correlation at position 3 (Chr18: 3,412,768; r_Spearman_ = 0.22, P = 0.02). Additionally, *TGIF1* mRNA levels in SAT exhibited a positive correlation with serum cholesterol (r_Pearson_ = 0.11, P < 0.001), remaining significant after correction for multiple testing. In OVAT, *TGIF1* expression showed a negative correlation with mean adipocyte diameter (r_Pearson_ = -0.2, P < 0.001) that remained significant after correction for multiple testing. Differences in mean adipocyte diameter between participants in the lowest and highest deciles of *TGIF1* expression in OVAT were statistically significant (Fig. 3C). Moreover, we observed a strong correlation, between mean adipocyte diameter in OVAT and blood DNAm of *TGIF1* for CpG-sites 1 (r_Spearman_ = 0.57, P = 0.007; Chr18: 3,412,695) and 3 (r_Spearman_ = 0.51, P = 0.01; Chr18: 3,412,768; Fig. 3D), with the same effect direction between mean adipocyte diameter in SAT and CpG-site 1 (Chr18: 3,412,695; r_Spearman_ = 0.48, P = 0.03). Serum resistin levels were associated with DNAm at site 1 (r_Spearman_ = 0.22, P = 0.05) and site 3 (r_Spearman_ = 0.3, P = 0.008). Anthropometric measurements such as waist circumference correlated significantly with DNAm at several sites, most notably at Position 2 (Chr18: 3,412,715; r_Spearman_ = 0.37, P < 0.001), which remained significant after multiple testing correction. Correlations analyses are depicted in Fig. 3E and described in detail in Supplemental Table 9 and 11.

### EIF5A and TGIF1 in childhood obesity

**Fig. 4:**
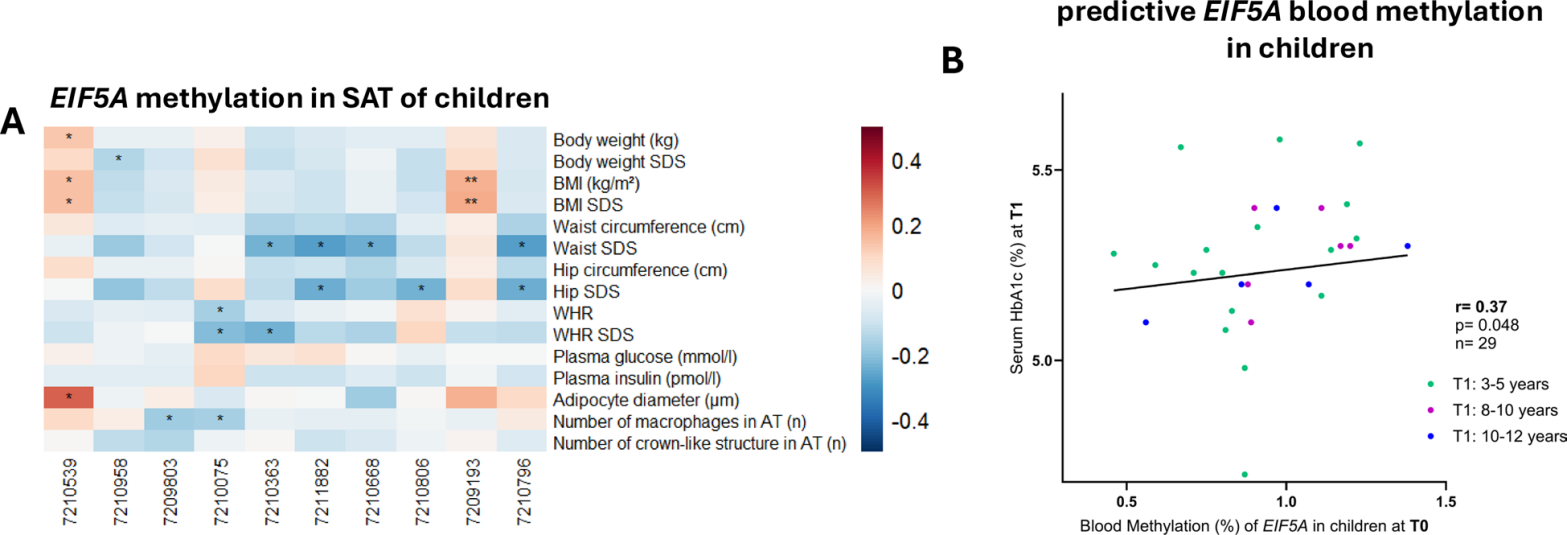
*EIF5A* regulation in children (**A**) Heatmap showing correlations between *EIF5A* DNAm (ß-values) in SAT (subcutaneous adipose tissue) and various clinical parameters in children of the Leipzig AT Childhood cohort at chosen CpG sites of the whole gene (EPICs) according to relevant correlations. CpG sites are named as positions in GRCh37. Clinical variables include body mass index (BMI), waist–hip ratio (WHR), Standard-deviation-score (SDS), and adipose-tissue (AT) measures. One asterisk (*) indicates significance at p < 0.05; two asterisks (**) indicate p < 0.01; three asterisks (***) indicates p < 0.001, with none of the correlations surviving Benjamini–Hochberg correction for multiple testing. Red indicates positive correlations and blue negative correlations, according to the legend. (**B**) Spearman correlation (r = 0.37) with linear regression line between *EIF5A* blood DNAm (%) analyzed in targeted pyrosequencing at a baseline timepoint and HbA1c (%) at later timepoints in children of the LIFE-Child cohort. The different time spans until HbA1c was measured, are indicated with green (3–5 years after measurement of DNAm), purple (8–10 years after) or blue (10–12 years after) dots in the graph. N = 29

Using an Illumina EPIC dataset (Illumina ID positions in GRCh37) from SAT of the Leipzig Adipose Tissue Childhood Cohort (N = 219) [43], we investigated the *EIF5A* DNAm status in children with and without obesity (Supplemental Table 7). We observed significantly higher methylation levels in children with obesity (2.39%) compared to those without obesity (2.18%, P = 0.02). However, this difference was confined to a single CpG site, cg7210539 (Chr17:7,307,220), which is located near the region identified in adults (cg7210196–cg7210280; Chr17:7,306,877–7,306,962). Across this and other CpG positions, we detected several correlations with obesity-related phenotypes, including BMI SDS, WHR, adipocyte diameter and macrophage count in SAT (all P < 0.05; Supplemental Table 12). Notably, only CpG site cg7210228 (Chr17:7,306,909) – which lies within the adult DMR – also showed a phenotype association, specifically with the number of crown-like structures in AT (r_Pearson_ = 0.15, P = 0.047). All correlations are shown in Fig. 4A and Supplemental Table 12.

**Fig. 5:**
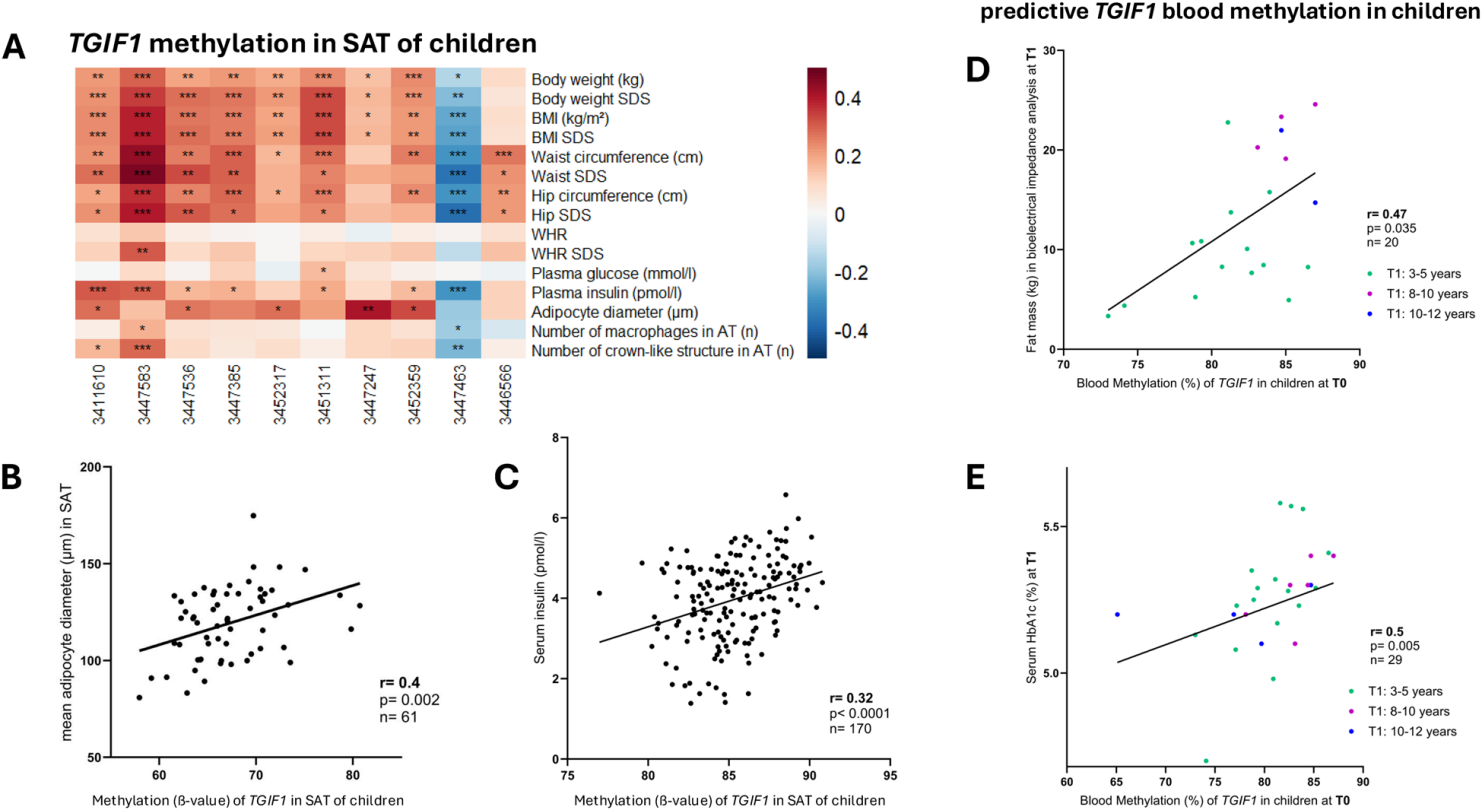
*TGIF1* regulation in children (**A**) Heatmap showing correlations between *TGIF1* DNAm (ß-values) in SAT (subcutaneous adipose tissue) and various clinical parameters in children of the Leipzig AT Childhood cohort at chosen CpG sites of the whole gene (EPICs) according to relevant correlations. CpG sites are named as positions in GRCh37. Clinical variables include body mass index (BMI), waist–hip ratio (WHR), Standard-deviation-score (SDS), and adipose-tissue (AT) measures. One asterisk (*) indicates significance at p < 0.05; two asterisks (**) indicate p < 0.01; three asterisks (***) indicates p < 0.001, with several of the correlations surviving Benjamini–Hochberg correction for multiple testing, namely the ones in CpG-position: 3,411,487, 3,411,610, 3,447,583, 3,452,443, 3,447,016, 3,447,536, 3,447,385, 3,411,821, 3,452,317, 3,455,229, 3,447,247, 3,452,359, 3,447,463, 3,446,566 and 3,449,374. Red indicates positive correlations and blue negative correlations, according to the legend. (**B**) Spearman correlation (r = 0.40) with linear regression line between *EIF5A* DNAm (ß-values) in subcutaneous adipose tissue (SAT) and the mean adipocyte diameter (µm) in SAT in children of the Leipzig AT Childhood cohort. N = 61. (**C**) Spearman correlation (r = 0.32) with linear regression line between *EIF5A* DNAm (ß-values) in subcutaneous adipose tissue (SAT) and the serum insulin levels (pmol/l) in children of the Leipzig AT Childhood cohort. N = 170. (**D**) Spearman correlation (r = 0.47) with linear regression line between *EIF5A* blood DNAm (%) analyzed in targeted pyrosequencing at a baseline timepoint and fat mass (kg) measured in bioelectrical impedance analysis at later timepoints in children of the LIFE-Child cohort. The different time spans until the fat mass was measured are indicated with green (3–5 years after measurement of DNAm), purple (8–10 years after) or blue (10–12 years after) dots in the graph. N = 20. (**E**) Spearman correlation (r = 0.50) with linear regression line between *EIF5A* blood DNAm (%) analyzed in targeted pyrosequencing at a baseline timepoint and HbA1c (%) at later timepoints in children of the LIFE-Child cohort. The different time spans until HbA1c was measured, are indicated with green (3–5 years after measurement of DNAm), purple (8–10 years after) or blue (10–12 years after) dots in the graph. N = 29

We expanded the DNAm analysis in SAT of this cohort also for *TGIF1* including the entire gene region. The most significant correlations identified in SAT of the Leipzig Adipose Tissue Childhood Cohort are presented in a heatmap in Fig. 5A (details in Supplemental Table 12). Across the gene, including 77 CpG sites, we identified CpG position cg3411487 (Chr18: 3,411,489; r_Pearson_ = 0.4, P = 0.002) and position cg3452359 (Chr18: 3,452,359; r_Pearson_ = 0.4, P = 0.001) showing positive associations to adipocyte diameter (Fig. 5B). Four CpG positions incl. e.g. cg3411610 (Chr18: 3,411,612) and cg3447583 (Chr18: 3,447,585; both r_Pearson_ = 0.3, P < 0.0001) show positive correlations with serum insulin levels (Fig. 5C). We also identified significant associations between *TGIF1* DNAm and the number of crown-like structures in AT at 2 CpG sites, after correction for multiple testing, which are namely cg3447583 (Chr18: 3,447,585; r_Pearson_ = 0.3, P = 0.0002) and cg3452443 (Chr18: 3,452,445; r_Pearson_ = 0.24, P = 0.002). Furthermore, DNAm levels also correlated with waist and hip circumferences individually, while no association with WHR could be observed.

### Obesity related trajectories of *EIF5A* and *TGIF1* DNA methylation from early childhood

To determine the predictive potential of DNAm for subsequent obesity and related metabolic outcomes – and to assess whether these epigenetic changes precede or follow weight gain – we analyzed blood DNAm in a longitudinal cohort of 75 children with normal weight at baseline (mean age: 8.6 years) who were re-assessed after an average of 7.8 years, by which time they had developed either underweight, normal weight, or overweight. For *EIF5A*, initial DNAm levels, in the blood of children with normal weight (average BMI SDS: -0.07, Supplemental Table 6), did not show changes according to their body weight status (underweight with BMI SDS: -1.6, normal weight with BMI SDS: -0.02 or overweight with BMI SDS: 1.6) at the second timepoint (average age: 16.4 years). Consistently, we did not detect an association between baseline DNAm levels and BMI at follow-up. However, we observed a correlation between the *EIF5A* DNAm at position 1 (Chr17: 7,306,877) and HbA1c levels at the second timepoint across all BMI SDS groups (r_Spearman_ = 0.37, P = 0.048) *(*Fig. 4B*)*.

Similarly, no differences in blood DNAm at the *TGIF1* locus were observed in children (BMI SDS and age as previously described for *EIF5A,* all shown in Supplemental Table 6) at the initial timepoint between those who were with underweight, normal weight or overweight at the second measurement nor was there an association between *TGIF1* DNAm and the BMI status at follow-up. However, we detected a positive correlation between baseline DNAm at position 1 (Chr18: 3,412,695) and total fat mass (r_Spearman_ = 0.47, P = 0.04), as well as HbA1c (r_Spearman_ = 0.5, P = 0.006) at the later follow-up timepoint (Fig. 5D and E, all in Supplemental Table 13).

To enhance clarity of the observed associations, a comprehensive summary of all *EIF5A* and *TGIF1* associations across tissues and phenotypes, including non-significant findings, is provided in Supplemental Fig. 1.

## Discussion

Gene methylation is an emerging target of research efforts regarding non-permanent genetic alterations that can be influenced by lifestyle and environment [14] in comparison to permanent changes like mutations or SNPs [10]. The search for accessible and reliable epigenetic biomarkers has been intensified in the field of metabolic diseases, as polygenic risk scores fall short in predictive power for complex phenotypes like obesity. In contrast to genetic risk scores, epigenetic markers can integrate both genetic susceptibility and environmental exposure [13, 61]. Epigenome-wide association studies have shown associations with several phenotypes related to adiposity [62–64], yet few studies have successfully identified and validated circulating DNAm biomarkers that could mirror AT function in obesity as diagnostic tool or render predictive potential. While recent large-scale deep learning–enabled GWAS have begun to elucidate the genetic architecture of adipocyte morphology and its links to cardiometabolic disease, knowledge regarding epigenetic precursors and regulatory mechanisms shaping adipocyte hypertrophy and dysfunction remains remarkably limited [65]. A major challenge in epigenomic biomarker discovery lies in distinguishing functionally relevant changes from background noise, particularly in the context of complex traits like obesity. To overcome this, we integrated transcriptomic and epigenomic datasets from AT and blood from metabolically well-characterized adult and pediatric cohorts and identified *EIF5A* and *TGIF1* with consistent variance in expression and DNA methylation across obesity stages within the included partly independent cohorts. *EIF5A* and *TGIF1* were hypermethylated in individuals with obesity and showed associations with metabolic and anthropometric traits, especially with altered glucose metabolism and adipocyte size. Additionally, *TGIF1* demonstrates stronger predictive potential, as its methylation levels in blood correlate with later metabolic outcomes in children. The regions analyzed in *EIF5A* and *TGIF1* lie within the gene’s promoter, a typical regulatory element with well-established links to transcriptional silencing [66] as shown in Fig. 2A (*EIF5A*) and 3A (*TGIF1*).

Recent studies highlighted the role of *EIF5A* in inflammatory signaling, particularly in macrophages residing in AT [67, 68]. In mouse models, altered *EIF5A* activation through DHPS inhibition (genetically as well as pharmacologically) led to comparable results of improved glucose tolerance [69, 70]. In our data, the blood-based DNAm of *EIF5A* increased progressively with BMI and showed pronounced differences between adults with normal weight and severe obesity. Expression of *EIF5A* was higher in OVAT than in SAT and was positively associated with both body fat percentage and hip circumference. Given that OVAT is characterized by higher endocrine activity and a stronger association to cardiometabolic diseases compared to SAT, these associations may implicate biological relevance.

This seems to be consistent with its proposed role in the immune-metabolic crosstalk, since increased *EIF5A* protein levels were observed in AT macrophages of obese mice [67] which is known to contribute to metabolic dysfunction [71]. Experimental evidence from murine models supports these findings, showing that inhibition of *EIF5A* reduced pro-inflammatory macrophage infiltration and improved glucose tolerance [67]. Importantly, in our study, the association between *EIF5A* methylation and HbA1c, a marker of long-time blood glucose, was particularly pronounced in individuals with T2D, suggesting a potential link to obesity-related metabolic dysfunction. The significantly higher expression of *EIF5A* in OVAT in comparison to SAT supports the hypothesis that its epigenetic regulation may mirror inflammatory and metabolic states, rather than adiposity per se* –* a notion consistent with evidence that adipocytes from different fat depots are most likely intrinsically distinct [72], even retaining characteristics when transplanted [73], with the depot of OVAT conferring a higher risk for metabolic complications[74]. This is further supported by correlations between DNAm and leucocyte counts, suggesting that changes in *EIF5A* DNAm in blood may partially reflect proinflammatory activity, especially considering that AT macrophages play a role in obesity-driven inflammation [71] and the inflammatory state of AT is associated with the clinical presentation of metabolic syndrome [75].

In children, correlations between the early-life *EIF5A* DNAm and metabolic parameters (HbA1c) in blood later in life were revealed. However, *EIF5A* may be considered a marker of metabolic inflammation, with only modest predictive value for prospective metabolic outcomes.

*TGIF1*, a transcriptional repressor of the TGF-β signaling pathway, has been shown to influence adipocyte differentiation [76]. Experimental disruption of *TGIF1* in preadipocytes reduces PPAR-γ expression and lipid accumulation, while insulin promotes *TGIF1* nuclear stability [76]. In our adult cohorts, *TGIF1* DNAm in blood and overall DNAm of *TGIF1* in OVAT [41], was higher in participants with obesity compared to controls with a BMI < 30 kg/m^2^, while *TGIF1* expression in OVAT negatively correlated with mean adipocyte diameter. These findings suggest a model in which increased methylation suppresses *TGIF1* expression, thereby impairing adipogenesis and promoting hypertrophic growth of existing adipocytes—hallmarks of metabolically unhealthy AT expansion and function. These findings may suggest a link of *TGIF1* regulation to metabolically unfavorable adipose remodeling. Interestingly, blood DNAm levels of *TGIF1* correlated with circulating resistin, which in humans is mainly produced by macrophages. Findings from humanized resistin transgenic mice and epidemiological studies indicate that human resistin may act as a mediator linking inflammation, insulin resistance, and atherosclerosis [77].

The potential of *TGIF1* as a biomarker was further supported by our findings in prospective pediatric cohorts. In the LIFE Child longitudinal study, blood was collected from normal weight children at an early age. Remarkably, baseline *TGIF1* DNAm predicted fat mass and HbA1c levels measured at a second time point (average age difference: 7.8 years). These findings may reflect the concept that epigenetic changes established early in life can shape future metabolic resilience or vulnerability and influence disease risk [26, 27]. In addition, *TGIF1* DNAm in SAT from children also correlated with serum insulin, further reinforcing the relevance of this gene to early adipose dysfunction. These results highlight the potential of *TGIF1* blood DNAm as a pre- or at least early diagnostic marker for metabolic dysfunction during obesity development. Consistent with our data, previous findings showed that blood mRNA levels of *TGIF1* increased following a polyphenol-enriched randomized controlled trial, alongside a reduction in body weight [78]. A marker based on adipocyte stress or impaired differentiation could guide early interventions beyond BMI. Our findings, together with existing literature, implicate a role of *TGIF1* in glucose metabolism and AT development—a fundamental mechanism likely underlying its high evolutionary conservation [79].

Several limitations should be noted. First, *TGIF1* and *EIF5A* were preselected based on known adiposity-related phenotypes, introducing potential bias. However, this targeted approach aligns with the study’s goal of identifying blood-measurable loci as accessible biomarkers. In LOBB, individuals with BMI < 30 kg/m^2^ were used as the comparison group rather than strictly lean controls, reflecting the cohort’s obesity-focused composition; however, adipose tissue metabolism may vary within this group, and more refined comparisons (e.g., BMI < 25 vs. obesity) were not feasible due to the limited number of lean participants. Although DNAm analysis was not genome-wide and may have overlooked other relevant regions, this focused design might enhance practicality for clinical application. In addition, the current study is a biomarker focused study and does therefor not include functional validation in adipose tissue, highlighting the need for future studies to directly assess the biological relevance of the identified loci in this context. Furthermore, validation in larger cohorts, the potential need for multi-marker approaches, and the identification of the most informative CpG sites remain important considerations for future research. Finally, the prognostic value of these markers remains to be established in future studies incorporating longitudinal data and matched tissue samples.

Second, although DNAm differences were statistically significant, their modest magnitude and narrow range limit their potential as standalone predictors. Predictive analyses in children were constrained by missing data, small sample size, and variability in baseline and follow-up time points, potentially reducing statistical power. Limitations in follow-up duration and tissue availability (blood vs. AT) further restricted these analyses. Moreover, in participants with T2D, HbA1c levels were measured under glucose-lowering medication, which may have influenced metabolic associations. However, as such medication typically lowers HbA1c values, any observed correlations might be more conservative than they would be in the absence of medication.

The generalizability of our findings remains to be confirmed in larger, independent cohorts, ideally integrating parallel methylation and expression profiling across matched tissues. Longitudinal studies with repeated sampling are needed to clarify the temporal dynamics and predictive value of *EIF5A* and *TGIF1* methylation in disease progression. Future research should employ single-cell epigenomics to resolve cell-type–specific effects and integrate multi-omics layers, including proteomics and metabolomics, to improve biomarker specificity and mechanistic insight.

## Conclusions

Our findings highlight the hypermethylation of *TGIF1* and *EIF5A* in individuals with obesity, each contributing differently to AT biology and metabolic regulation. *EIF5A* appears to serve primarily as a diagnostic marker, reflecting obesity-associated inflammation, while *TGIF1* demonstrates additional predictive value, particularly in children, where early alterations in DNAm (before onset of obesity) were associated with future AT dysfunction and metabolic risk. These results support continued efforts aimed at exploration of the role of epigenetic signatures in the stratification of patients suffering from obesity and related metabolic complications.

## Supplementary Information

Supplementary Material 1

Supplementary Material 2

Supplementary Material 3

## Data Availability

All data generated or analyzed during this study are included in this published article and its Supplementary information files. Previously published datasets analyzed in this study are available at the Health Atlas portal (https://www.health-atlas.de/studies/57) for the LIFE-Adult study (HumanMethylation850 BeadChip array and RNA-seq data), and at the ArrayExpress database at EMBL-EBI (https://www.ebi.ac.uk/arrayexpress) under accession number E-MTAB-13564 for the LOBB study (HumanMethylation850 BeadChip array data). Access to the human transcriptome data from the LOBB study is regulated by the LOBB steering committee and requires an approved Data Use Agreement (DUA) outlining the permitted research purposes and protections for participant privacy. For access, contact Matthias Blüher (matthias.blueher@medizin.uni-leipzig.de) or Anne Hoffmann (anne.hoffmann@helmholtz-munich.de).

## Abbreviations

ALAT: Alanine aminotransferase
ASAT: Aspartate aminotransferase
AT: Adipose tissue
AU: Arbitrary units
BIA: Bioelectrical impedance analysis
BMI: Body mass index
CRP: C-reactive protein
DNAm: DNA methylation
DMR: Differentially methylated region
EPIC: Illumina Infinium MethylationEPIC BeadChip
FPG: Fasting plasma glucose
FPI: Fasting plasma insulin
fT3: Free triiodothyronine
fT4: Free thyroxine
gGT: Gamma-glutamyl transferase
HbA1c: Glycated hemoglobin A1c
HDL: High-density lipoprotein
HOMA-IR: Homeostatic model assessment of insulin resistance
IL-6: Interleukin-6
IGT: Impaired glucose tolerance
LDL: Low-density lipoprotein
LOBB: Leipzig Obesity BioBank
OVAT: Omental-visceral adipose tissue
oGTT2h: 2-Hour oral glucose tolerance test
PCR: Polymerase chain reaction
RNA-seq: RNA sequencing
SAT: Subcutaneous adipose tissue
SDS: Standard deviation score
T0: Baseline time point
T1: Follow-up time point
T2D: Type 2 diabetes mellitus
TSH: Thyroid-stimulating hormone
TSS: Transcription start site
WBC: White blood cells
WHR: Waist–hip ratio

## Gene names

HSD11B1L: Hydroxysteroid 11-beta dehydrogenase 1-like
C15orf40: Chromosome 15 open reading frame 40
SULT4A1: Sulfotransferase family 4A member 1
CSNK1D: Casein kinase 1 delta
CEP76: Centrosomal protein 76
DMRT2: Doublesex and mab-3 related transcription factor 2
SESN3: Sestrin 3
EEF1A1: Eukaryotic translation elongation factor 1 alpha 1
LRRCC1: Leucine rich repeat and coiled-coil domain containing 1
HSPA1A: Heat shock protein family A member 1A
ART3: ADP-ribosyltransferase 3
TP63: Tumor protein p63
PCGF3: Polycomb group ring finger 3
ZBTB20: Zinc finger and BTB domain containing 20
SGMS1: Sphingomyelin synthase 1
WNT7B: Wingless-type MMTV integration site family member 7B
LOC145783: Uncharacterized LOC145783
SHANK1: SH3 and multiple ankyrin repeat domains 1
EMX2OS: Empty spiracles homeobox 2 opposite strand RNA
PCDHGA8: Protocadherin gamma subfamily A member 8
PI16: Peptidase inhibitor 16
TUBB: Tubulin beta class I
VTI1A: Vesicle transport through interaction with t-SNAREs 1A
CLPB: Caseinolytic peptidase B homolog
FARP1: FERM, ARH/RhoGEF and pleckstrin domain protein 1
LRCH3: Leucine rich repeat and calponin homology domain containing 3
HDAC4: Histone deacetylase 4
FRYL: FRY-like transcription coactivator
ITPR2: Inositol 1,4,5-trisphosphate receptor type 2
REV3L: REV3-like DNA directed polymerase zeta catalytic subunit
DST: Dystonin
PTPRG: Protein tyrosine phosphatase receptor type G
NDRG2: N-myc downstream regulated gene 2
PPP2CA: Protein phosphatase 2 catalytic subunit alpha
UBE2D4: Ubiquitin conjugating enzyme E2 D4
LDHA: Lactate dehydrogenase A
TEP1: Telomerase-associated protein 1
TTC22: Tetratricopeptide repeat domain 22
CCDC140: Coiled-coil domain containing 140
C1QTNF6: C1q and TNF-related protein 6
ECE2: Endothelin converting enzyme 2
GNAI2: Guanine nucleotide-binding protein G(i) subunit alpha 2
EIF5A: Eukaryotic translation initiation factor 5A
MRGPRF: MAS-related G protein-coupled receptor member F
TGIF1: TGFB-induced factor homeobox 1
STK19: Serine/threonine kinase 19
C3orf22: Chromosome 3 open reading frame 22
CDH22: Cadherin 22
SRCIN1: SRC kinase signaling inhibitor 1
PCDHGB2: Protocadherin gamma subfamily B member 2
PCDHGB3: Protocadherin gamma subfamily B member 3
RASA3: RAS p21 protein activator 3
DNAJB6: DnaJ heat shock protein family member B6
GMDS: GDP-mannose 4,6-dehydratase
FAF1: Fas associated factor 1
SND1: Staphylococcal nuclease domain containing 1
DNHD1: Dynein heavy chain domain containing 1
BTBD9: BTB domain containing 9
LOC101929710: Uncharacterized LOC101929710
ZZEF1: Zinc finger ZZ-type and EF-hand domain containing 1
LINC00536: Long intergenic non-protein coding RNA 536
