# Supplementary figures and images for: Blood DNA methylation of EIF5A and TGIF1 is associated with adipose tissue health and metabolic outcomes in obesity: a multi-cohort study
Source: Clin Epigenetics. 2026 Jun 16;18:118. doi: 10.1186/s13148-026-02177-y (PMC13274240; doi:10.1186/s13148-026-02177-y)

**Supplemental Figure 1**


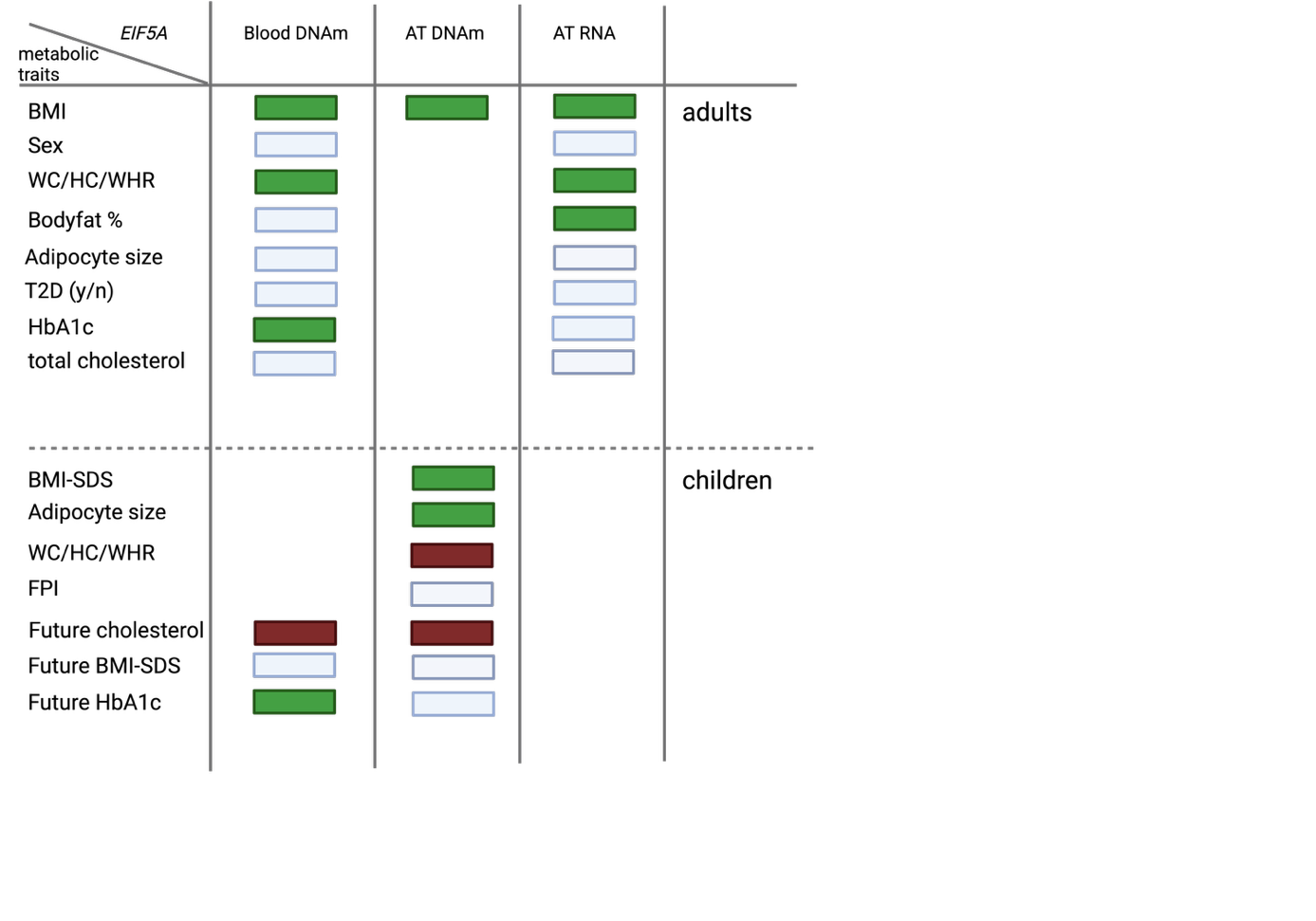
A


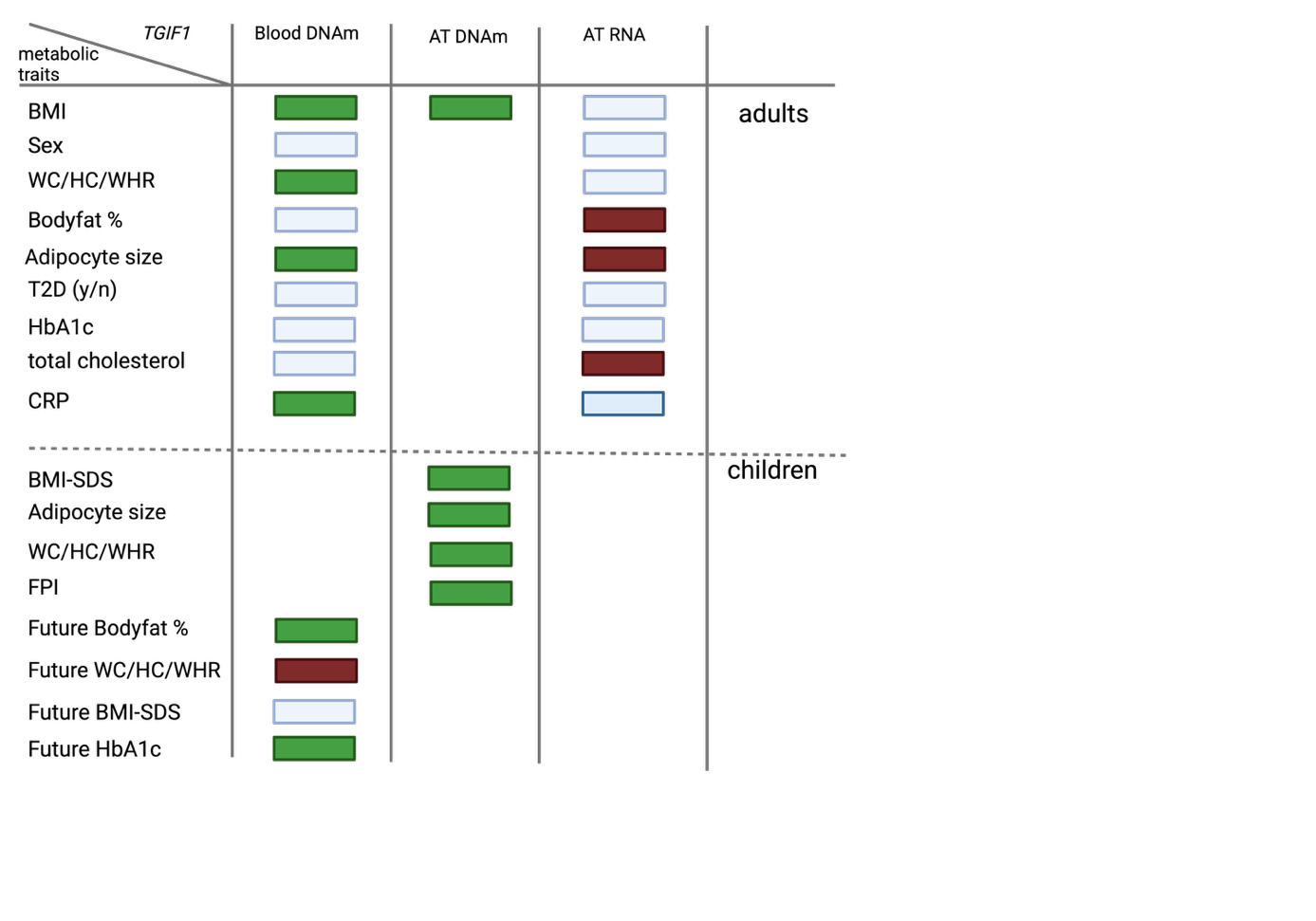
B

Supplement: Supplementary file 1 — Supplementary Material 1 [file 13148_2026_2177_MOESM1_ESM.docx]
