# Supplementary material for: Blood DNA methylation of EIF5A and TGIF1 is associated with adipose tissue health and metabolic outcomes in obesity: a multi-cohort study
Source: Clin Epigenetics. 2026 Jun 16;18:118. doi: 10.1186/s13148-026-02177-y (PMC13274240; doi:10.1186/s13148-026-02177-y)
Supplement: Supplementary file 3 — Supplementary Material 3 [file 13148_2026_2177_MOESM3_ESM.docx]

**Supplements**

**Supplemental Figure 1**

**Summary of Significant and Non-Significant Gene Associations**

Overview of biomarker associations for *EIF5A*  (A) and *TGIF1* (B), including the analyzed omics layer (DNA methylation or mRNA expression), tissue type, and study cohort (adults or children). For each association, the corresponding trait or phenotype is shown. Effect directions are color-coded: green indicates a significant positive effect, red indicates a significant negative effect, and grey denotes a non-significant effect. Empty fields indicate analyses that were not available. Details on statistical tests and significance thresholds are provided in the Methods section.

**Supplemental Table 1. Clinical characteristics of the LIFE-Adult cohort with genome-wide DNA methylation analyses in blood**

Clinical and anthropometric characteristics of participants from the LIFE-Adult cohort included in the genome-wide DNA methylation analyses (ß-values in EPICs) used for the candidate gene selection process. Participants were stratified into non-obese and obese groups. Data are presented as *N*, mean, and standard deviation (SD). Group comparisons were performed using unpaired Student’s *t*-tests. Nominally significant differences (*P* < 0.05) are highlighted in bold. (**Abbreviations:** BMI, body mass index; FPG, fasting plasma glucose; HbA1c, glycated hemoglobin A1c; HDL, high-density lipoprotein; LDL, low-density lipoprotein; WHR, waist–hip ratio.)

**Supplemental Table 2. Clinical characteristics of LOBB participants with RNA sequencing data in adipose tissue**

Clinical, anthropometric, and metabolic characteristics of participants from the Leipzig Obesity BioBank (LOBB) with adipose tissue mRNA expression data (log₂ VST), which were used for the candidate gene selection process and subsequent expression analyses. Participants were stratified into non-obese, obese, and severely obese groups. Data are presented as *N*, mean, and standard deviation (SD). Group comparisons were performed using one-way analysis of variance (ANOVA) to test for equality of means. Nominally significant differences (*P* < 0.05) are highlighted in bold. (**Abbreviations:** ALAT, alanine aminotransferase; ASAT, aspartate aminotransferase; BMI, body mass index; CRP, C-reactive protein; FPG, fasting plasma glucose; FPI, fasting plasma insulin; fT3, free triiodothyronine; fT4, free thyroxine; gGT, gamma-glutamyl transferase; HbA1c, glycated hemoglobin A1c; HDL, high-density lipoprotein; HOMA-IR, homeostatic model assessment of insulin resistance; IL-6, interleukin-6; LDL, low-density lipoprotein; oGTT2h, 2-hour oral glucose tolerance test; TSH, thyroid-stimulating hormone; WHR, waist–hip ratio.)

**Supplemental Table 3. Clinical characteristics of LOBB participants with genome-wide promoter DNA methylation data in adipose tissue**

Clinical and anthropometric characteristics of participants from the LOBB with genome-wide promoter DNA methylation data (hyper- and hypo-methylation per promoter, MeDIP on GeneChip Human Promoter) in adipose tissue, which were used in the candidate gene selection process. Participants were stratified into non-obese and obese groups. Data are presented as *N*, mean, and standard deviation (SD). Group comparisons were performed using unpaired Student’s *t*-tests. Nominally significant differences (*P* < 0.05) are highlighted in bold. (**Abbreviations:** BMI, body mass index; FPG, fasting plasma glucose; HbA1c, glycated hemoglobin A1c; HDL, high-density lipoprotein; IL-6, interleukin-6; LDL, low-density lipoprotein; TSH, thyroid-stimulating hormone; WHR, waist–hip ratio.)

**Supplemental Table 4. Clinical characteristics of LOBB participants undergoing bariatric surgery**

Clinical and metabolic characteristics of LOBB participants undergoing two-step bariatric surgery with available DNA methylation data (ß-values in EPICs) from blood, subcutaneous adipose tissue (SAT), and omental/visceral adipose tissue (OVAT). These data were used as part of the candidate gene selection process. Values are presented as *N*, mean and standard deviation (SD) AU. Differences between the paired probes before and after the bariatric surgery were assessed using the non-parametric Wilcoxon signed-rank test. Nominally significant differences (*P* < 0.05) are highlighted in bold. (**Abbreviations:** ALAT: alanine aminotransferase; ASAT: aspartate aminotransferase; BMI: body mass index; CRP: C reactive protein; FPG: fasting plasma glucose; FPI: fasting plasma insulin; gGT: gamma-glutamyl transferase; HbA1c: glycated haemoglobin A1c; HDL: high density lipoprotein; HOMA-IR: homeostatic model assessment for insulin resistance; LDL: low density lipoprotein.)

**Supplemental Table 5. Clinical characteristics of adult participants included in the blood DNA methylation validation analyses**

Clinical, anthropometric, and metabolic characteristics of adult participants from the LOBB included in the blood DNA methylation validation analyses (%) of *EIF5A* and *TGIF1* in targeted pyrosequencing. Group comparisons were performed using one-way analysis of variance (ANOVA) to test for equality of means across BMI-defined groups stratified into non-obese, obese and severe obese. Data are presented as N, mean, and standard deviation (SD). Nominally significant differences (*P* < 0.05) are highlighted in bold. (**Abbreviations:** ALAT, alanine aminotransferase; ASAT, aspartate aminotransferase; BMI, body mass index; CRP, C-reactive protein; FPG, fasting plasma glucose; FPI, fasting plasma insulin; fT3, free triiodothyronine; fT4, free thyroxine; gGT, gamma-glutamyl transferase; HbA1c, glycated hemoglobin A1c; HDL, high-density lipoprotein; HOMA-IR, homeostatic model assessment of insulin resistance; IL-6, interleukin-6; LDL, low-density lipoprotein; oGTT2h, 2-hour oral glucose tolerance test; TSH, thyroid-stimulating hormone; WHR, waist–hip ratio.)

**Supplemental Table 6. Phenotypic characteristics of LIFE Child participants with blood DNA methylation validation of *EIF5A* and *TGIF1***

Clinical, anthropometric, and metabolic characteristics of children from the LIFE Child cohort included in the blood DNA methylation validation analyses (%), measured with targeted pyrosequencing, of *EIF5A* and *TGIF1*. Baseline (T0) values are reported for children with normal weight, and follow-up (T1) values are reported for children who developed underweight, normal weight, or overweight. Data are presented as *N*, mean, and standard deviation (SD). Group comparisons were performed using one-way analysis of variance (ANOVA). Nominally significant differences (*P* < 0.05) are highlighted in bold. (**Abbreviations:** BIA, bioelectrical impedance analysis; BMI, body mass index; FPG, fasting plasma glucose; FPI, fasting plasma insulin; HbA1c, glycated hemoglobin A1c; HDL, high-density lipoprotein; LDL, low-density lipoprotein; SDS, standard deviation score; WHR, waist–hip ratio; WBC, white blood cells. Asterisk (*) indicates values referring only to the follow-up timepoint (T1).)

**Supplemental Table 7. Clinical characteristics of the Leipzig Adipose Tissue Childhood cohort with genome-wide DNA methylation in subcutaneous adipose tissue**

Clinical, anthropometric, and metabolic characteristics of children from the Leipzig Adipose Tissue Childhood cohort with genome-wide DNA methylation (DNAm) data (ß-values in EPICs) in subcutaneous adipose tissue (SAT), stratified into non-obese and obese groups. Data are presented as *N*, mean, and standard deviation (SD). Group comparisons were performed using unpaired Student’s *t*-tests. Nominally significant differences (*P* < 0.05) are highlighted in bold. (**Abbreviations:** BMI, body mass index; FPG, fasting plasma glucose; FPI, fasting plasma insulin; SDS, standard deviation score; WHR, waist–hip ratio; SAT, subcutaneous adipose tissue.)

**Supplemental Table 8. Candidate genes identified through the multi-step selection process**

Overview of 62 candidate genes identified through the integrative multi-step selection process combining blood DNA methylation data and adipose tissue gene expression data. Differential DNA methylation was assessed separately in promoter and gene body regions, and differential gene expression was analyzed independently in subcutaneous adipose tissue (SAT) and omental visceral adipose tissue (OVAT). Genes are grouped according to tissue (SAT or OVAT), genomic context (promoter or gene body), and direction of regulation in individuals without obesity compared to individuals with obesity, indicating concordant upregulation or downregulation of DNA methylation and gene expression.

**Supplemental Table 9. Associations between adipose tissue gene expression and clinical characteristics of Adults in the Leipzig Obesity BioBank**

Correlation analyses between mRNA expression levels (log₂ VST) of 12 candidate genes in adipose tissue and anthropometric, metabolic, inflammatory, and adipose tissue–specific phenotypes in adult participants of the LOBB. Correlations are reported separately for SAT and OVAT. Pearson correlation coefficients (*r*) and corresponding *P*-values are shown. Nominally significant associations (*P* < 0.05) are highlighted in bold, and associations remaining significant after Benjamini–Hochberg correction for multiple testing are indicated by asterisks. (**Abbreviations:** BMI, body mass index; CRP, C-reactive protein; FPG, fasting plasma glucose; FPI, fasting plasma insulin; HbA1c, glycated hemoglobin A1c; HDL, high-density lipoprotein; HOMA-IR, homeostatic model assessment of insulin resistance; IL-6, interleukin-6; LDL, low-density lipoprotein; oGTT2h, 2-hour oral glucose tolerance test; SAT, subcutaneous adipose tissue; OVAT, omental (visceral) adipose tissue; WHR, waist–hip ratio.)

**Supplemental Table 10. Primer sequences used for targeted bisulfite sequencing**

Primer sequences used for targeted bisulfite sequencing (pyrosequencing) of the *EIF5A* and *TGIF1* promoter regions in the validation cohorts. The table includes primer names, genomic target regions, primer sequences (5′–3′), amplicon lengths, and corresponding genomic coordinates. Primer design was based on the candidate differentially methylated regions identified in the selection process and corresponds to regions previously detected by EPIC array analyses. All primers were designed to specifically amplify bisulfite-converted DNA.

**Supplemental Table 11. Associations between blood DNA methylation of *TGIF1* and *EIF5A* and clinical characteristics in the LOBB validation cohort**

Correlation analyses between blood DNA methylation levels (%), measured with targeted pyrosequencing, of *TGIF1* and *EIF5A* and anthropometric, metabolic, inflammatory, and adipose tissue–related clinical characteristics in adults of the LOBB validation cohort. Spearman correlation coefficients (*rₛ*) and corresponding *P*-values are shown. Pearson partial correlation coefficients adjusted for age, sex, and body mass index (BMI) are presented in italics. Nominally significant associations (*P* < 0.05) are highlighted in bold and marked with one asterisk (*). Associations remaining significant after correction for multiple testing are indicated by two asterisks (**). (**Abbreviations:** BMI, body mass index; CRP, C-reactive protein; FPG, fasting plasma glucose; FPI, fasting plasma insulin; fT3, free triiodothyronine; fT4, free thyroxine; HbA1c, glycated hemoglobin A1c; HDL, high-density lipoprotein; HOMA-IR, homeostatic model assessment of insulin resistance; IL-6, interleukin-6; LDL, low-density lipoprotein; oGTT2h, 2-hour oral glucose tolerance test; OVAT, omental (visceral) adipose tissue; SAT, subcutaneous adipose tissue; T2D, type 2 diabetes mellitus; TSH, thyroid-stimulating hormone; WHR, waist–hip ratio.)

**Supplemental Table 12. Associations between adipose tissue DNA methylation of *TGIF1* and *EIF5A* and clinical characteristics in the Leipzig Adipose Tissue Childhood Cohort**

Correlation analyses between genome-wide DNA methylation (DNAm) levels (ß-values in EPICs) of *TGIF1* and *EIF5A* in adipose tissue and anthropometric, metabolic, and inflammatory phenotypes in children from the Leipzig Adipose Tissue Childhood Cohort. CpG site positions are reported according to GRCh37. CpG sites previously analyzed in blood of children and adults are indicated in italics. Pearson correlation coefficients (*r*) and corresponding *P*-values are shown. Nominally significant associations (*P* < 0.05) are highlighted in bold and marked with one asterisk (*), and associations remaining significant after correction for multiple testing are indicated by two asterisks (**). (**Abbreviations:** BIA, bioelectrical impedance analysis; BMI, body mass index; CRP, C-reactive protein; FPG, fasting plasma glucose; FPI, fasting plasma insulin; fT3, free triiodothyronine; fT4, free thyroxine; HbA1c, glycated hemoglobin A1c; HDL, high-density lipoprotein; HOMA-IR, homeostatic model assessment of insulin resistance; IL-6, interleukin-6; LDL, low-density lipoprotein; oGTT2h, 2-hour oral glucose tolerance test; OVAT, omental (visceral) adipose tissue; SAT, subcutaneous adipose tissue; SDS, standard deviation score; TSH, thyroid-stimulating hormone; T2D, type 2 diabetes mellitus; WHR, waist–hip ratio.)

**Supplemental Table 13. Associations between blood DNA methylation of *TGIF1* and *EIF5A* and clinical characteristics in children of the LIFE Child cohort at follow-up timepoint**

Correlation analyses between blood DNA methylation (DNAm) levels (%) in targeted pyrosequencing of *TGIF1* and *EIF5A* and anthropometric, metabolic, and inflammatory characteristics in children from the LIFE Child cohort at follow-up timepoint. CpG sites correspond to those analyzed in the adult validation cohort. Spearman correlation coefficients (*rₛ*) and corresponding *P*-values are shown. Nominally significant associations (*P* < 0.05) are highlighted in bold and marked with one asterisk (*). Associations remaining significant after correction for multiple testing are indicated by two asterisks (**). (**Abbreviations:** BIA, bioelectrical impedance analysis; BMI, body mass index; CRP, C-reactive protein; FPG, fasting plasma glucose; FPI, fasting plasma insulin; fT3, free triiodothyronine; fT4, free thyroxine; HbA1c, glycated hemoglobin A1c; HDL, high-density lipoprotein; HOMA-IR, homeostatic model assessment of insulin resistance; IL-6, interleukin-6; LDL, low-density lipoprotein; oGTT2h, 2-hour oral glucose tolerance test; OVAT, omental (visceral) adipose tissue; SAT, subcutaneous adipose tissue; SDS, standard deviation score; TSH, thyroid-stimulating hormone; T2D, type 2 diabetes mellitus; WHR, waist–hip ratio.)

**Supplemental Appendix – Detailed Description of the Multi-Step Gene Selection Process**

To identify robust blood-based DNAm markers that distinguish individuals with obesity from those without obesity, and that also reflect molecular regulation within adipose tissue, we implemented a structured, multi-stage selection process (schematically shown in Figure 1). This approach progressively narrowed a genome-wide set of candidates to a small number of biologically relevant genes suitable for targeted validation.

Step 1: Initial identification of candidate genes across blood DNAm and adipose-tissue transcriptomics

In the first step, we integrated two independent datasets:

1. Adipose-tissue expression data (SAT and OVAT) from the LOBB cohort
2. Blood DNAm data from the LIFE-Adult cohort.

Both datasets were analyzed to identify differential gene expression (in adipose tissue) and differential DNAm (in blood) between individuals with and without obesity. For DNAm, promoter and gene body regions were evaluated independently. For gene expression, SAT and OVAT were analyzed separately. This resulted in four parallel analyses, each yielding sets of genes or promoter with significant obesity-related differences at adj. P < 0.01. These lists were then overlaid. Promoter and genes exhibiting both differential methylation in blood and differential expression in AT were retained as initial candidates. Because each of the four datasets (promoter or gene body region in either SAT or OVAT) could furthermore yield up- or downregulation, the analytical combinations produced 16 theoretical regulatory patterns. For each pattern, we selected the top five genes (where available), prioritizing those with the largest methylation difference between obese and non-obese individuals, followed by the magnitude of expression differences. This procedure resulted in 62 candidate genes (Supplemental).

Step 2: Replication of candidate genes DNAm in AT

In the second stage, we examined whether these 62 genes also displayed differential DNAm directly within adipose tissue. To this end, we analyzed our previously published genome-wide methylation dataset from a subset of the LOBB cohort, in which DNAm was measured in SAT and OVAT. Genes showing consistent differential DNAm in AT were advanced to the next step, yielding 10 candidate genes.

Step 3: Replication of candidate gene DNAm in blood and AT of weight-loss data

To assess whether DNAm of these 10 genes responds to metabolic improvement, we next evaluated DNAm changes in a longitudinal dataset from a subset of LOBB participants who underwent two-step bariatric surgery, with methylation measured in blood, SAT, and OVAT before and after weight loss. Six genes showed significant DNAm alterations following weight-loss surgery and were thus considered robust markers of metabolic state. These genes were:

- *TGIF1* (TGFB induced factor homeobox 1)
- *EIF5A* (Eukaryotic translation initiation factor 5A)
- *FAF1* (Fas associated factor 1)
- *ZBTB20* (Zinc finger and BTB domain containing 20)
- *DST* (Dystonin)
- *HDAC4* (Histone deacetylase 4)

Step 4: Associations with metabolic phenotypes in AT

To refine this list further, we investigated associations between AT mRNA levels of these six genes and metabolic and anthropometric traits in LOBB participants (N=1554). Only correlations which remained significant after correction for multiple testing using the Benjamini-Hochberg procedure were considered (Figure 1; Supplemental Table 2).

Notably, in OVAT, *EIF5A* and *TGIF1* were the only genes that demonstrated significant associations with phenotypes beyond general adiposity measures (BMI and body fat percentage), a criterion essential for prioritizing clinically meaningful targets. Given that OVAT exhibits higher endocrine activity and a stronger link to cardiometabolic complications than SAT, the associations observed for these two genes further underscore their biological relevance. Previous studies support the functional importance for both genes: *TGIF1* knockdown in 3T3-L1 preadipocytes impairs adipocyte differentiation, whereas *EIF5A* undergoes a unique post-translational modification via deoxyhypusine synthase (DHPS), required for its activation, which may make *EIF5A* particularly relevant in the context of epigenetic regulation, as DNAm alterations may influence its activation state and downstream processes.

Based on the combined evidence from cross-tissue regulation, responsiveness to weight loss, phenotype associations, and biological plausibility, *EIF5A* and *TGIF1* emerged as the strongest candidates for functional follow-up. Regions for targeted bisulfite sequencing were selected according to previously identified differentially methylated regions that responded to bariatric surgery in blood and AT. In Step 1, both *EIF5A* and *TGIF1* were identified as hypermethylated and transcriptionally downregulated in individuals with obesity, prompting a focused analysis of their promoter regions. The genomic regions prioritized for validation were located on chromosome 17 (GRCh38: 7,306,877–7,306,962) for *EIF5A* and chromosome 18 (GRCh38: 3,412,695–3,412,785) for *TGIF1*
